# The Influence of Self-Regulation Behaviors on University Students’ Intentions of Persistance

**DOI:** 10.3389/fpsyg.2019.02284

**Published:** 2019-10-10

**Authors:** Ana Bernardo, María Esteban, Antonio Cervero, Rebeca Cerezo, Francisco Javier Herrero

**Affiliations:** Department of Psychology, University of Oviedo, Oviedo, Spain

**Keywords:** higher education, university, university dropout, academic success, academic self-regulation

## Abstract

The implementation of the European higher education area (EHEA) is a true paradigm change in university education in which the student, with particular consideration given to autonomous work, takes the place of the teacher as the central element of the teaching-learning process. In this autonomous work, the strategies the students regularly use become particularly important, given the supposition that doing that work will lead to academic success. The objective of this study is to analyze the variables that influence students’ expectations of success, measured through their intention to persist on the course they are doing. A questionnaire designed *ad hoc* was given to a sample of 1037 university students. It included aspects related to reasons for choosing the course, institutional integration, use of self-regulation strategies, and intention to drop out. Data analysis allowed the identification of satisfaction with the course chosen and appropriate study skills acquired in secondary education as predictors of expectations of academic persistance, with some differences in terms of gender. Other strategies such as class attendance or going deeply into course content did not figure. These results are at odds with the principles underlying the EHEA and show that they have not yet been interiorized by the students, who continue to perceive their studies more traditionally.

## Introduction

The continuous, rapid technological, and social advances in the last fifty years have led to the new social paradigm of the “knowledge society” (Pérez et al., 2018), basing economic growth on people’s intellectual capital. It seeks to improve citizen education and training, making the most of people’s capacity for continuous learning, producing better qualified individuals, and so improving the number and quality of jobs available.

Universities play a fundamental role in this context, as they are the prime bodies for the production of knowledge through scientific research, transmission of knowledge through education and training, and diffusion of knowledge by different channels (Comisión de las Comunidades Europeas, 2003).

If we add to that the growing process of globalization, it is no surprise that in education at the European level there is a plan for convergence that would allow universities to join forces, and unite educational policies. This has given us the European higher education area (EHEA), with the objective of modernizing higher education teaching and institutions across Europe (Alonso-Sáez and Arandia-Loroño, 2017).

The European Higher Education Area is not only about structural and organizational change, but rather a real paradigm shift with implications in the way we understand the teaching-learning process (Esteve and Gisbert, 2011) which affects institutions at all levels: economic, methodological, social, and evaluational. However, various studies have highlighted that neither students nor teachers, nor the institutions themselves, are adequately prepared or equipped with the means to properly enact this change in educational paradigm (López et al., 2015; Alonso-Sáez and Arandia-Loroño, 2017).

One of the most significant changes is the consideration of the student as the central element of the teaching-learning process. The teacher, up to now the fundamental pillar of teaching from the more behavioral point of view, cedes ground to the student, who is established as an autonomous, self-regulated learner. So students are the protagonist, responsible for their own educational process, in line with the constructivist paradigm (Conole, 2013; Muñoz-Cantero and Mato-Vásquez, 2014; Tirado-Morueta and Aguaded-Gómez, 2014).

This autonomous character, present in the educational tenets of the EHEA, was most fully realized in the adoption of the European credit transfer system (ECTS) as the unit of measure for academic credit. ECTS credit system gives importance to classroom activities but also take into account offsite activities. So, for example, a subject with 6 ECTS credits will include in the plan 60 h of classroom work and 90 h of autonomous student work, making up the 150 actual hours of work in the subject, as generally 1 ECTS credit equals 25 hours of effective student work (Art. 4. Boletín Oficial del Estado [BOE], 2003). Boletín Oficial del Estado [BOE] (2003) stated in its explanatory preamble that this system was a conceptual reformulation of higher education curricula via the adoption of new teaching models focusing on student work. It also defined the extent to which theoretical and practical teaching would be incorporated, as well as other academic activities students were required to carry out to reach the learning objectives in each of the subjects of syllabuses (Art. 3. Boletín Oficial del Estado [BOE], 2003).

In this context, educational quality is a principal aim for European Higher Education institutions. In this sense, quality is mainly assed in terms of graduation rates, quality of instruction and excellence of research (European Association for Quality Assurance in Higher Education [ENQA], 2015). Thus, student dropout is a great problem that in Europe reach rates between 20 and 40% of university students (Vossensteyn et al., 2015). Since academic performance has showed to be the main predictor of university dropout it is important to extend the research about it (Gairín et al., 2014; Soria-Barreto and Zúñiga-Jara, 2014; Cerezo et al., 2015), particularly in the new EHEA context.

Academic performance is a fundamental variable in student progress in an institution (Casanova et al., 2018), especially in the early stages of adapting to the university system. Literature clearly shows the huge number of variables that can influence student performance, and those that may be subject to intervention have been the object of particular study, for instance psycho-educational variables such as prior training, study habits and interest or engagement in the course. In addition, the level of prior knowledge is an academic variable which is generally related to performance, especially when this knowledge is insufficient or inadequate as the basis for new learning (Soria-Barreto and Zúñiga-Jara, 2014; López et al., 2016). In fact, the influence of this variable in later academic performance in university is so great that researchers such as Miranda et al. (2013) note it as a highly influential variable and the prime institutional variable influencing students’ academic failure.

Knowledge and application of appropriate study techniques have also been shown to directly influence the decision to continue with a course of study (Arriaga et al., 2011; Tuero et al., 2018), as has satisfaction with the chosen program (Bethencourt et al., 2008). Academic success requires not only a good choice of program, a good base level of knowledge and adequate study methods, it also requires regular study. Daily or periodic study is another widely researched variable related to academic performance and success (García, 2014; Bakker et al., 2015; Cerezo et al., 2016). This study engagement is easier when the student is interested in the content (Ordóñez and Rodríguez, 2015; Garrote et al., 2016) and so, indicators such as more in-depth personal study of course content contribute significantly to successfully completing subjects and programs (Carbonero et al., 2013).

Most of these variables are indicators of self-regulation of learning (de la Fuente et al., 2017). Hence, in the new European educational paradigm self-regulation of learning is encouraged in order to promote academic success and persistance (Álvarez and López, 2011). In fact, the EHEA assigns a prime role to self-regulation strategies because of their influence on the teaching-learning process and on academic results. However, it seems paradoxical that despite personal autonomy and learning-skill acquisition being part of the Spanish educational curriculum in primary (Boletín Oficial del Estado [BOE], 2014) and secondary Education (Boletín Oficial del Estado [BOE], 2015), a large proportion of students at university fail when facing the demands of self-regulation of learning (Gil-Flores, 2015; Cerezo et al., 2017; Klemenčič, 2017). This is not exclusive to Spain, it is an international problem, both in traditional and virtual environments (Broadbent and Poon, 2015; Trevors et al., 2016).

Faced with this, it is worth asking ourselves whether the cause may be found in a lack of preparation (in terms of prior knowledge or study habits) or whether it is a consequence of a discrepancy between students’ perceptions of study requirements and reality, or an insufficient understanding of those requirements. In the context of the EHEA, variables that are traditionally considered to be influential in academic performance and success, such as regular class attendance, gain particular importance, as the indications teachers gather from students in those sessions are essential to orient autonomous work, as demonstrated in research by Bernardo et al. (2015); Esteban et al. (2016), and Muñoz-Cantero and Mato-Vásquez (2014).

The aim of this study is to analyze the influence of the variables outlined above on expectations of academic persistence. A better fit between prior achievement and subsequent achievement may function as a predictor of satisfaction with results and continuation with the course of study (Khattab, 2015; Velázquez and González, 2017).

To that end, the objective of this study is to examine the possible influence that study habits and personal baggage may have on students’ expectations of their academic success and persistence on the institution. Specifically, we aim to see whether those variables related to the implementation of EHEA are perceived by students as precursors of satisfactory academic progress and persistence. Thus, we draw two hypothesis:

H1: There will be higher expectations of persistence, in those students who consider their prior training (in terms of prior knowledge of and mastery of study techniques) to be sufficient to the demands of the course that they are on.
H2: The students will consider those variables related to self-regulated learning behaviors important for they academic persistence.

## Materials and Methods

### Sample

The sample was made up of 1037 first-year students in the University of Oviedo. The majority (73.9%) were women, and the average age was 19.94 years old (*SD* = 4.17). The sampling method used was non-probability intentional selection, based on the working-group teachers’ access to the sample.

The students were doing various undergraduate degree courses. The most common were primary education (22.6% of the students), nursing (22.2%), infant education (16.9%), and psychology (12%). Students were doing other degree courses to a lesser extent (less than 10% of students on each course): Economics; Law; Law, Management and Business Administration (double degree^1^); English; Chemistry; Speech Therapy; Physics; Physics and mathematics (double degree^1^); and business and marketing.

### Instruments

An *ad hoc* questionnaire was created for data collection in this study about university experience, self-regulation strategies applyed in higher education, dropout intentions and reasons for dropping out of university (Tuero et al., 2018). It had a Cronbach alpha of 0.79.

It was made up of eleven classification variables and many other variables grouped in eight dimensions. The classification variables refer to factors such as: identifying data, sex, age, availability of grants, branch of secondary education, final secondary education grade, university entrance exam grade, mother’s educational qualifications, father’s educational qualifications, whether they are doing subjects in the first course they enrolled on, whether it is their first chosen degree, whether they do any paid work and if so, their working hours, and whether they do any non-curricular activities outside class-time and if so, what type of activity and how long they spend on it (sports, academic or social activities, paid work, etc).

The rest of the questionnaire corresponded to 8 dimensions that contain 66 items about: (1) reason for choosing the program; (2) prior knowledge; (3) finances; (4) current situation; (5) interest in the program; (6) integration; (7) institutional variables; and (8) self-regulation strategies.

Apart from the classification variables, which were dichotomous, multiple choice or open response questions, the responses for the remaining dimensions were via a five-point Likert-type scale with the following scoring: (1) completely disagree; (2) disagree; (3) neither agree nor disagree; (4) agree; and (5) completely agree.

### Procedure

The questionnaire process began initially with contact with teachers who were signed up to a university teaching innovation project, This teaching innovation project sought to analyze the motivations behind drop-out intentions and university students’ self-regulation strategies.

The questionnaires were administered, on paper, in the classroom to be completed in the teachers’ own classes by freshmen, 3 months after starting the course. This was to allow an evaluation before the first exams in the school year.

The procedure include written consent of participation in the study and agree with the criteria stablished by our university ethics committee.

### Data Analysis

In order to examine the possible relationships between student self-regulatory behavior and expectations of academic persistance we ask students about their persistence intentions. Thus, through students’ intentions to continue on the course that they started, we looked into students expectations of success. We used categorical regression techniques to evaluate the impact that the variables described previously could have on the probability that a student would stay on their current course or drop out.

Independent variables included in the analysis were categorical so we applied a categorical regression model were students expectations of persist on the program was the dependent variable and there were nine independent variables; prior knowledge, adequacy of prior acquired study techniques, interest in study, satisfaction with the chosen program, class attendance, daily study, interest in course content, performance orientation, and deepen in course contents.

Data analysis were performed using the IBM SPSS Statistics v.24 package.

## Results

The categorical regression model was applied first, given that it is the best fit to the mix of ordinal Likert-type variables making up the questionnaire and the dichotomized criterion variable. This model explains 22.3% of the variance in the participants’ expectations of remaining on their current program.

The analysis of variance of the model, which is significant (*p* < 0.005), ensure its validity [*F*(21) = 15.0713447].

Only two variables significantly contribute to the model: the opinion that study techniques used to date are adequate, and satisfaction with the choice of program. Table 1 shows that satisfaction is more important (*B* = 0.364, *p* < 0.005) than positive opinions about proper study techniques (*B* = 0.106, *p* < 0.005).

**TABLE 1:** Regression coefficients for students’ expectations.

	**Standardized coefficients**		**df**	***F***	**Sig.**
				
	***B***	**Estimation of sample simulation (1000) of standard error**			
I feel that what I learned previously in secondary school is sufficient to deal with this first university year	0.098	0.063	1	2.431	0.119
I think that the study techniques used up to now have been adequate	0.106	0.036	2	8.806	0.000
I am more interested in studying now than I was in secondary school	0.047	0.060	2	0.616	0.540
I feel satisfied with my choice of program	0.364	0.044	3	68.924	0.000
I have good attendance, I attend most of the classes in the university	0.093	0.067	2	1.938	0.144
I keep up to date with my subjects	–0.051	0.046	3	1.229	0.298
I am very interested in the program content	0.081	0.056	2	2.103	0.122
I try to get the best marks possible	0.059	0.039	4	2.211	0.066
I look into the topics we deal with in class in order to know more about the subject	–0.049	0.063	2	0.605	0.546

We found statistically significant differences in the first predictor (related to students’ appropriate use of study techniques), such that those students who were thinking of dropping out tended to respond more negatively to the item (completely disagree and disagree) than those who were not thinking of dropping out. This means that using appropriate study techniques prevent students’ intentions of droping out. Nonetheless, in regard to IBM (2019) the effect size of this variable is small (χ^2^ = 30.865; df = 4; *p* < 0.000; VCramer = 0.173).

Similarly, with the second predictor (satisfaction with choice of program), there were also significant differences. Students who were considering dropping out were less satisfied with their choices (completely disagree, disagree, and neither agree nor disagree) than those who were not thinking of dropping out (completely agree). Following the guidelines of IBM (2019) we can cathegorize the effect size as higher than for the other predictor, in the moderate effect range (χ2 = 206.108; df = 8; *p* < 0.000;VCramer = 0.446).

Following the results in the contingency tables, we carried out a correspondence analysis to visualize where the differences lay, including the gender variable. We found that while differences in the variable about study techniques were inter-gender and the differences in the satisfaction variable were intra-gender.

For the first variable (use of appropriate study techniques) we found the values shown in Table 2, where the variability would be almost completely explained by a single dimension, with an inertia of 0.41 out of 0.45 (91.9%).

**TABLE 2:** Summary of correspondence analysis: Study techniques vs. sex.

**Dimension**	**Singular value**	**Inertia**	**Chi-squared**	**Sig.**	**Proportion of inertia**
1	0.203	0.041			0.919
2	0.049	0.002			0.054
3	0.035	0.001			0.027
Total		0.045	46.523	0.000^∗^	1.000

*^∗^Df = 12, *N* = 1037.*

**FIGURE 1:**
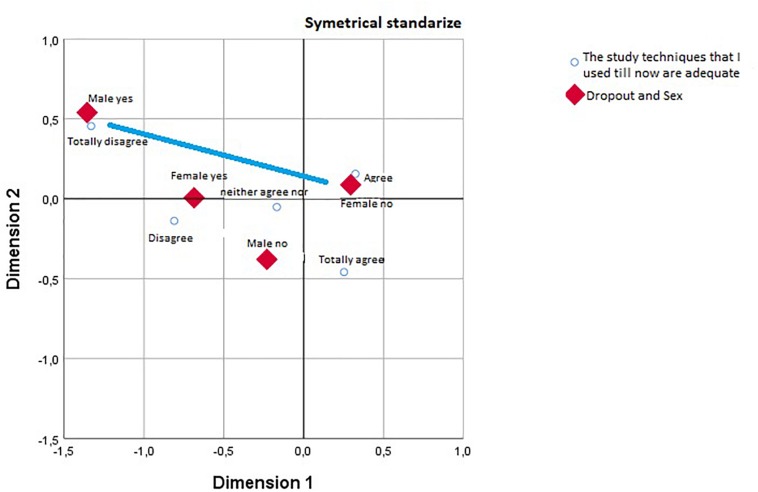
Study techniques vs. sex.

Figure 1 shows how men who are thinking of dropping out are associated with low evaluations of their use of study techniques in contrast to women who are not thinking of dropping out, who tend to score them as adequate.

With the second variable (satisfaction with the chosen degree), the results are shown in Table 3. As with the previous case, the variability is mostly explained by a single dimension, with an inertia of 0.21 out of 0.23 (88.1%).

**TABLE 3:** Summary of correspondence analysis: Satisfaction with choice of program vs. sex.

**Dimension**	**Singular value**	**Inertia**	**Chi-squared**	**Sig.**	**Proportion of inertia**
1	0.458	0.041			0.881
2	0.147	0.002			0.091
3	0.082	0.001			0.028
Total		0.045	46.523	0.000^∗^	1.000

*^∗^Df = 12, *N* = 1037.*

**FIGURE 2:**
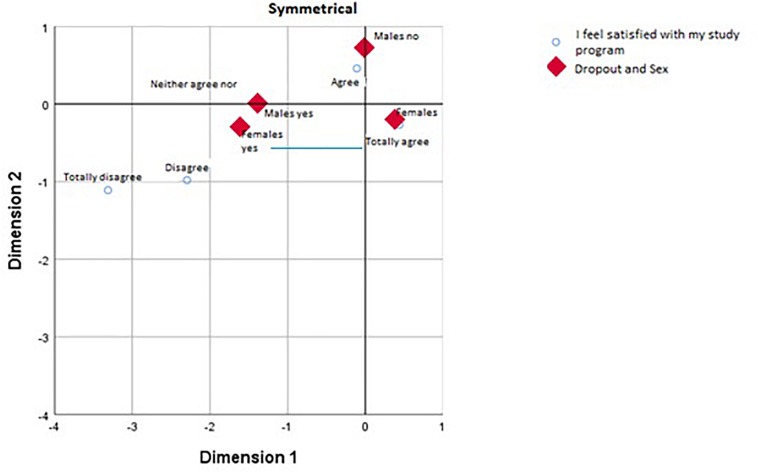
Satisfaction with program choice vs. sex.

As Figure 2 shows, women who are thinking of dropping out are associated with values of completely disagree and disagree when it comes to satisfaction with their choice of course, whereas women who are not thinking of dropping out give more positive evaluations (completely agree).

## Conclusion

The process of transition from secondary education to university is not an easy one for students, as it requires adaptation to an unknown, demanding environment regardless of what they might have been taught in prior educational and guidance processes. In this context, academic performance and expectations surrounding it are particularly interesting variables (Stinebrickner and Stinebrickner, 2014; Wolters and Hussain, 2015; Honicke and Broadbent, 2016).

In particular, in the European context, the EHEA brings along the requirement for students to develop an autonomous learning (Boletín Oficial del Estado [BOE], 2003). Thus, it is important to know whether the students understand the obligations that they need to match in order to accomplish this goal (McCardle et al., 2017).

Therefore, in this study we analyzed those variables which, according to student expectations, influence academic persistance. Thus, we assumed that it will be those expectations which can condition their behavior for proper performance.

Our results provide evidence, in line with research in this field, of the importance students place on study techniques, an indicator which is widely related to satisfactory achievement (Navarro et al., 2015; Ng, 2018). In our case, we did not confirm the weight given to prior knowledge, in contrast to other research (Albalate et al., 2011; Roksa et al., 2017). Having prior knowledge and study techniques depends on the itinerary of prior studies (Martínez et al., 2016). Hence, the results confirm our first hypothesis, but only partially, demonstrating the need to ensure that students starting different programs do so by the appropriate selection of a specific, individualized academic itinerary (Álvarez and López, 2017; Tuero et al., 2017). This would lead to have an appropriate prior knowledge and to have acquired appropriate study techniques, which in turn would lead students to have higher expectations of persistence (Bennett, 2003).

It does seem paradoxical that student’s perceptions and expectations of persistence are not related to other variables of significant learning and self-regulation, which does not support our second hypothesis. EHEA sift the educational paradigm, giving more protagonism to the student, who is supposed to be an autonomous learner. This is particularly important to bear in mind that when planning subjects, as can be seen in any teaching guide that follows the premises of EHEA, one must consider not only classroom activities such as lectures, practical classes, laboratory classes and tutorial groups, but also non-classroom activities such as individual and team work that occasionally require more time, and always the added need to learn autonomously and with self-regulation (Art. 4. Boletín Oficial del Estado [BOE], 2003).

Thus, some of the variables that we have studied – like class attendance or daily study- are important to succeed in the EHEA (Tomlinson, 2017). Despite that, our results are consistent with other studies and show how the participants do not feel that these variables are important with regard to achieving satisfactory academic success. So, variables as fundamental as interest in the subject being studied (Ghasemi and Dowlatabadi, 2018), more in-depth personal study of course content (Montes, 2012; Bogarín et al., 2018), and academic engagement in terms of attendance or being up to date with work (Cerezo et al., 2017; Rissanen, 2018) are not perceived as important for success by students, in opposition to EHEA principles. Since these variables are indicators of the three dimensions of learning self-regulation of learning -motivational, behavioral, and cognitive-, we can conclude that students do not consider important to be a self-regulated learner.

These results seem to show that the postulates that gave rise to the creation and implementation of the EHEA, particularly the ECTS system of credits, have not yet been interiorized by students, who continue to perceive their study more traditionally. It is necessary to continue improving effective interventions regarding learning self-regulation; in this sense training programs in higher education such eTRAL (Cerezo et al., 2017) or Metatutor (Bouchet et al., 2016) have shown to have positive impacts on academic performance and success (Esteban et al., 2017) and can encourage better fit between students’ characteristics and the requirements from EHEA based study plans.

Finally, future research should be aimed at increasing sample heterogeneity in different university years to understand whether these results apply to other programs or knowledge areas and whether there are significant differences between them. Intervention policies may be proposed to provide a better student guidance, able to guarantee a better adjustment to the context and demands of EHEA.

## Data Availability Statement

The datasets generated for this study are available on request to the corresponding author.

## Ethics Statement

The studies involving human participants were reviewed and approved by the Secretaría General de la Universidad de Oviedo. The patients/participants provided their written informed consent to participate in this study.

## Author Contributions

All authors contributed to the writing of the manuscript and development of the research. AB organized the sample gathering and designed the research instrument. ME and RC helped to design the research instrument and gather the sample. AC and FH coded the data and performed the analysis.

## Conflict of Interest

The authors declare that the research was conducted in the absence of any commercial or financial relationships that could be construed as a potential conflict of interest.

## Footnotes

1 There are several double degrees in the University of Oviedo, where pupils study two different programs at the time.
